# Liraglutide exhibits potential anti-tumor effects on the progression of intrahepatic cholangiocarcinoma, in vitro and in vivo

**DOI:** 10.1038/s41598-024-64774-2

**Published:** 2024-06-14

**Authors:** Ronnakrit Trakoonsenathong, Waritta Kunprom, Chaiwat Aphivatanasiri, Padcharee Yueangchantuek, Paslada Pimkeeree, Supannika Sorin, Kullanat Khawkhiaw, Ching-Feng Chiu, Seiji Okada, Sopit Wongkham, Charupong Saengboonmee

**Affiliations:** 1 Cho-Kalaphruek Excellent Research Project for Medical Students, Faculty of Medicine, Khon Kaen University, Khon Kaen, Thailand; 2 Department of Biochemistry, Faculty of Medicine, Khon Kaen University, Khon Kaen, 40002 Thailand; 3 Department of Pathology, Faculty of Medicine, Khon Kaen University, Khon Kaen, Thailand; 4 Faculty of Medical Sciences, Naresuan University, Phitsanulok, Thailand; 5 Cholangiocarcinoma Research Institute, Khon Kaen University, Khon Kaen, Thailand; 6 Graduate Institute of Metabolism and Obesity Sciences, Taipei Medical University, Taipei, Taiwan; 7 Division of Hematopoiesis, Joint Research Center for Human Retrovirus Infection, Kumamoto University, Kumamoto, Japan

**Keywords:** Cholangiocarcinoma, Diabetes mellitus, Glucagon-like peptide 1 receptor, Liraglutide, Biochemistry, Cancer, Cell biology, Molecular biology, Gastroenterology, Molecular medicine, Oncology

## Abstract

Glucagon-like peptide 1 receptor (GLP-1R) agonist is an emerging anti-diabetic medication whose effects on the risk and progression of cholangiocarcinoma (CCA) are controversial. This study aimed to elucidate the roles of GLP-1R and its agonists on intrahepatic CCA (iCCA) progression. Expressions of GLP-1R in iCCA tissues investigated by immunohistochemistry showed that GLP-1R expressions were significantly associated with poor histological grading (*P* = 0.027). iCCA cell lines, KKU-055 and KKU-213A, were treated with exendin-4 and liraglutide, GLP-1R agonists, and their effects on proliferation and migration were assessed. Exendin-4 and liraglutide did not affect CCA cell proliferation in vitro, but liraglutide significantly suppressed the migration of CCA cells, partly by inhibiting epithelial-mesenchymal transition. In contrast, liraglutide significantly reduced CCA tumor volumes and weights in xenografted mice (*P* = 0.046). GLP-1R appeared downregulated when CCA cells were treated with liraglutide in vitro and in vivo. In addition, liraglutide treatment significantly suppressed Akt and STAT3 signaling in CCA cells, by reducing their phosphorylation levels. These results suggested that liraglutide potentially slows down CCA progression, and further clinical investigation would benefit the treatment of CCA with diabetes mellitus.

## Introduction

Cholangiocarcinoma (CCA) is a malignancy emerging at any point of the biliary tract^1^. According to the 8th Edition American Joint Committee on Cancer Staging for Hepato-pancreato-biliary Cancer, CCAs are classified as intrahepatic, perihilar, and distal types^2^. The highest global incidence of CCA has been reported in the Northeast of Thailand^3,4^. Liver cancer, including CCA, is the most common cancer among Thai males, while it is the second cause of death from cancer nationwide^5^. Many risk factors are associated with CCA development, including hepatitis B, hepatitis C, obesity, diabetes mellitus (DM), smoking, and alcohol use^6,7^. However, *Opisthorchis viverrini* (*Ov*) infection has been recognized as the most significant risk factor for CCA in Thailand. The different risk factors for CCA contribute to the variation in genetic profiles and pathogenesis of CCA in each region of the world^8–11^. Therefore, a specific understanding of CCA carcinogenesis and its associated risk factors in a defined type and region will benefit the precise prevention and treatment.

Although *Ov* infection is strongly associated with CCA in Thailand, only approximately 1% of *Ov*-infected patients develop CCA^12^. Considering other risk factors for CCA, DM is striking. Almost all Thailand regions with high liver cancer mortality rates geographically overlap with those of DM^12,13^. Furthermore, about 60% of the patients who underwent CCA resection at our center had fasting blood glucose levels in prediabetic (100–125 mg/dL) or diabetic ranges (≥ 126 mg/dL)^14^. A previous cross-sectional study has demonstrated that the combination of *Ov* infection and DM strengthened the risks of CCA development more than either *Ov* infection or DM alone^15^. DM is, therefore, an emerging risk factor for CCA development and probably plays a key role in CCA carcinogenesis in Thailand, where *Ov* infection is also endemic.

Diabetic hyperglycemia can induce growth and metastatic activities of CCA cells in vitro via multiple pathways, i.e., signal transducer and activator of transcription 3 (STAT3)^16^, nuclear factor-kappa B (NF-κB)^17^, and glycogen synthase kinase 3/β-catenin pathways^18^. In addition, metformin, the first-line therapy for DM type 2, exerts anti-proliferative and anti-metastatic activities against CCA cells by targeting STAT3 and NF-κB^19^ and enhancing chemotherapeutic sensitivity of CCA to cisplatin, the standard chemotherapy^20^. On the other hand, a cohort study in the United Kingdom showed that incretin-based therapies, including dipeptidyl peptidase-4 (DPP-4) inhibitor and glucagon-like peptide 1 receptor (GLP-1R) agonists, were associated with an increased risk of CCA in adults with type 2 DM^21^. Discordantly, independent case–control studies in Italy^22^ and Scandinavia^23^ have shown a null effect of incretin-based medications and the increased risk of CCA development. Paradoxical results on the effects of GLP-1R agonists on CCA were also reported. Although exendin-4 (exenatide), a GLP-1R agonist, could increase chemosensitivity and inhibit the proliferation of CCA cells^24^, the GLP-1R itself promoted epithelial-mesenchymal transformation (EMT), resulting in enhanced migration and invasion of CCA^25^. Further, increased GLP-1R expressions were reported in the bile duct epithelium in response to obstructive biliary tract, and activation of GLP-1R promoted proliferation and anti-apoptosis of cholangiocytes^26^. Thus, roles of GLP-1R and its agonists in CCA remain to be elucidated, not only for the appropriate management of DM in those patients but also to provide the opportunity for repurposing anti-diabetic drugs for CCA treatment^27^.

Incretin-based therapies (DPP-4 inhibitors and GLP-1R agonists) have been widely used as a second or third-line treatment for type 2 DM nowadays due to their benefits for other associated complications^28^. However, the pro- and anti-tumor effects of incretin-based drugs on CCA development and progression are still controversial and inconclusive. Thus, the present study aimed to determine the clinicopathological significance of GLP-1R in CCA using tumor tissues from patients in *Ov*-endemic regions and to investigate the roles of liraglutide, a GLP-1R agonist widely used in patients with DM. A better understanding and clarification of the effects of this drug on CCA would be very helpful for the management of DM in patients with CCA as well as general DM patients in *Ov*-endemic areas.

## Results

### Patients characteristics

A total of 30 patients with iCCA were retrospectively included in this study. All patients underwent surgical resection for CCA removal at Srinagarind Hospital, Khon Kaen University, Thailand. The patients were composed of 19 men (63.30%) and 11 women (36.70%). The median age was 56 years (range, 37–79 years). Patients with preoperative fasting blood glucose (FBG) ≥ 126 mg/dL were classified as having DM and hyperglycemia, and those with FBG < 126 mg/dL were classified as non-DM, according to the diagnostic criteria recommended by the American Diabetes Association^29^. Other demographic and clinicopathological data is shown in supplementary Table S1.

### GLP-1R expressions were associated with poor histological grading

**Figure 1:**
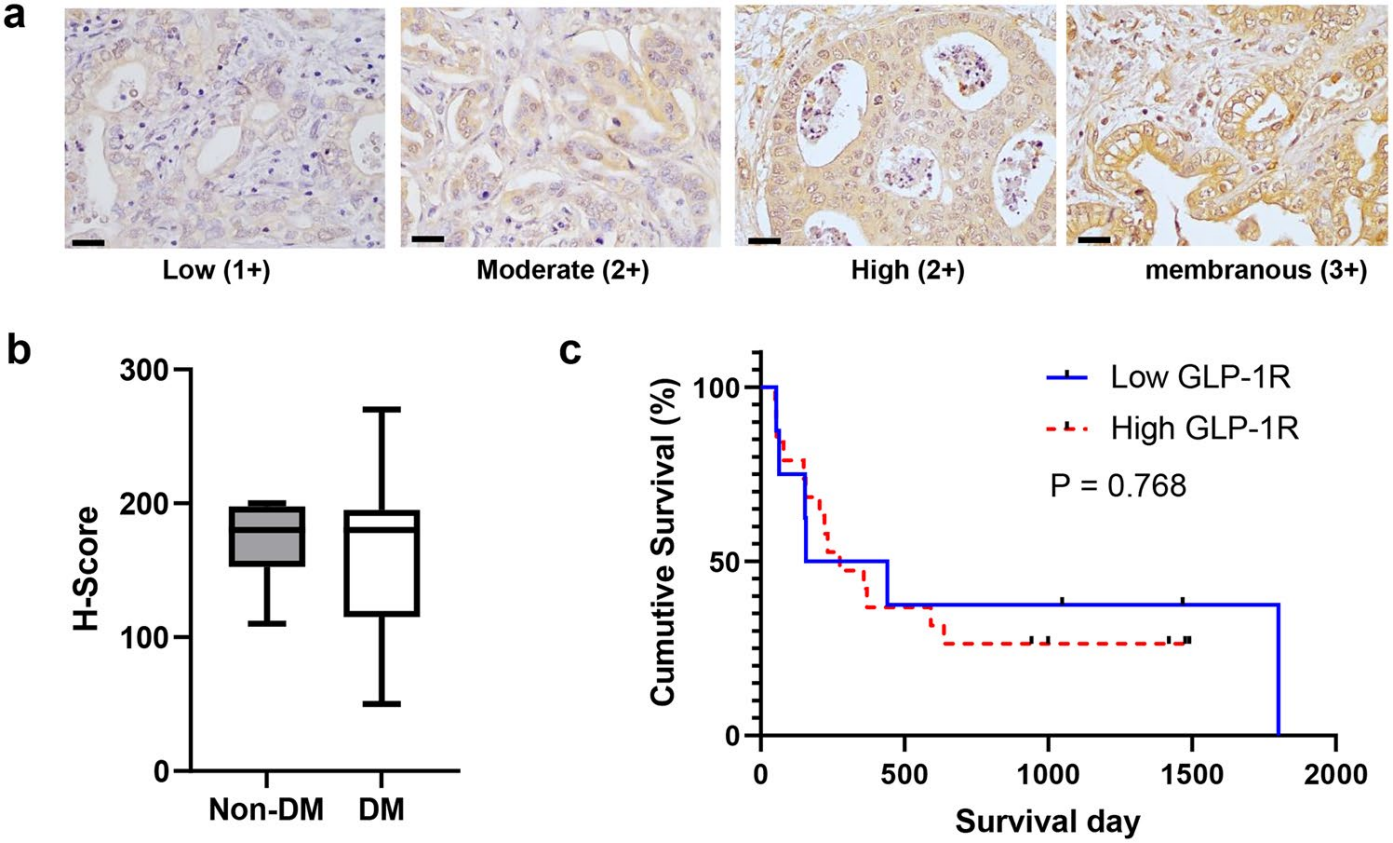
GLP-1R expression in iCCA tumor tissues. (**a**) The figures represent the scoring of GLP-1R intensity in iCCA tissues, only the membranous staining pattern was defined as 3 + as it suggests the function of the membrane-bound receptor. (**b**) GLP-1R expressions in CCA tumor tissues are not different for patients with or without diabetes mellitus (Student’s *t*-test). (**c**) The expression of GLP-1R is not associated with the overall survival of patients with iCCA (Log-rank test). Scale bars represent 20 µm.

The H-scores of GLP-1R expressions ranged from 10 to 270, with a normal distribution of the data (*P* > 0.05, Shapiro–Wilk test) and an arithmetic mean of 142.70 ± 8.47. Thus, the mean H-score (H = 143) was used as the cut-off point for high or low expression of GLP-1R in iCCA tumor tissues. Based on the assumption that GLP-1R agonist was used as anti-diabetic medication among patients with DM and that these patients may experience the effects of GLP-1R agonist, we compared the levels of GLP-1R expression between patients with and without DM. The expression levels of GLP-1R were, however, not different between DM and non-DM groups (Fig. 1a, b). Regardless of DM status, high GLP-1R expression was significantly associated with poor histological grading of iCCA (*P* = 0.027). However, GLP-1R expression was not associated with cumulative overall survival of patients (*P* = 0.768, log-rank test) (Fig. 1c). The analyses for associations of GLP-1R expressions and clinicopathological data are shown in Table 1. Due to the limited number of included CCA, which might confound statistical analyses, the present study then mainly focuses on molecular mechanisms of the involvements of GLP-1R and its agonists on the progression of CCA using the in vitro and in vivo models.

**Table 1:** Univariate analysis of GLP-1R expression and clinicopathological characteristics of iCCA.

Clinicopathological characteristics and laboratory parameters	GLP1R expression		*P* value (2-sided)
	High (H-score ≥ 143)	Low (H-score < 143)	
*Age (years)*			
$\ge$ 56	8	13	0.443
< 56	5	4	
*Sex*			
Male	12	9	0.419
Female	7	2	
*Diabetic status*			
DM	9	4	0.645
Non-DM	10	2	
*Histological grading*			
Well differentiation	7	9	0.027*
Moderately differentiation	6	0	
Poorly differentiation	2	0	
*Vascular invasion*			
Positive	16	4	> 0.999
Negative	7	2	
*Regional lymph node metastasis*			
Positive	7	12	> 0.999
Negative	3	6	
*Tumor size (longest diameter)*			
≥ 4 cm	15	4	0.646
< 4 cm	6	3	
*CA19-9 (IU/mlL)*			
> 37	3	4	0.293
$\le$ 37	4	1	
*CEA (ng/mL)*			
> 5.00	1	2	0.091
$\le$ 5.00	7	4	
*TNM staging*			
I	3	5	> 0.999
II–IV	6	12	

*P < 0.05.

### GLP-1R agonists did not affect the proliferation of CCA cells in vitro

**Figure 2:**
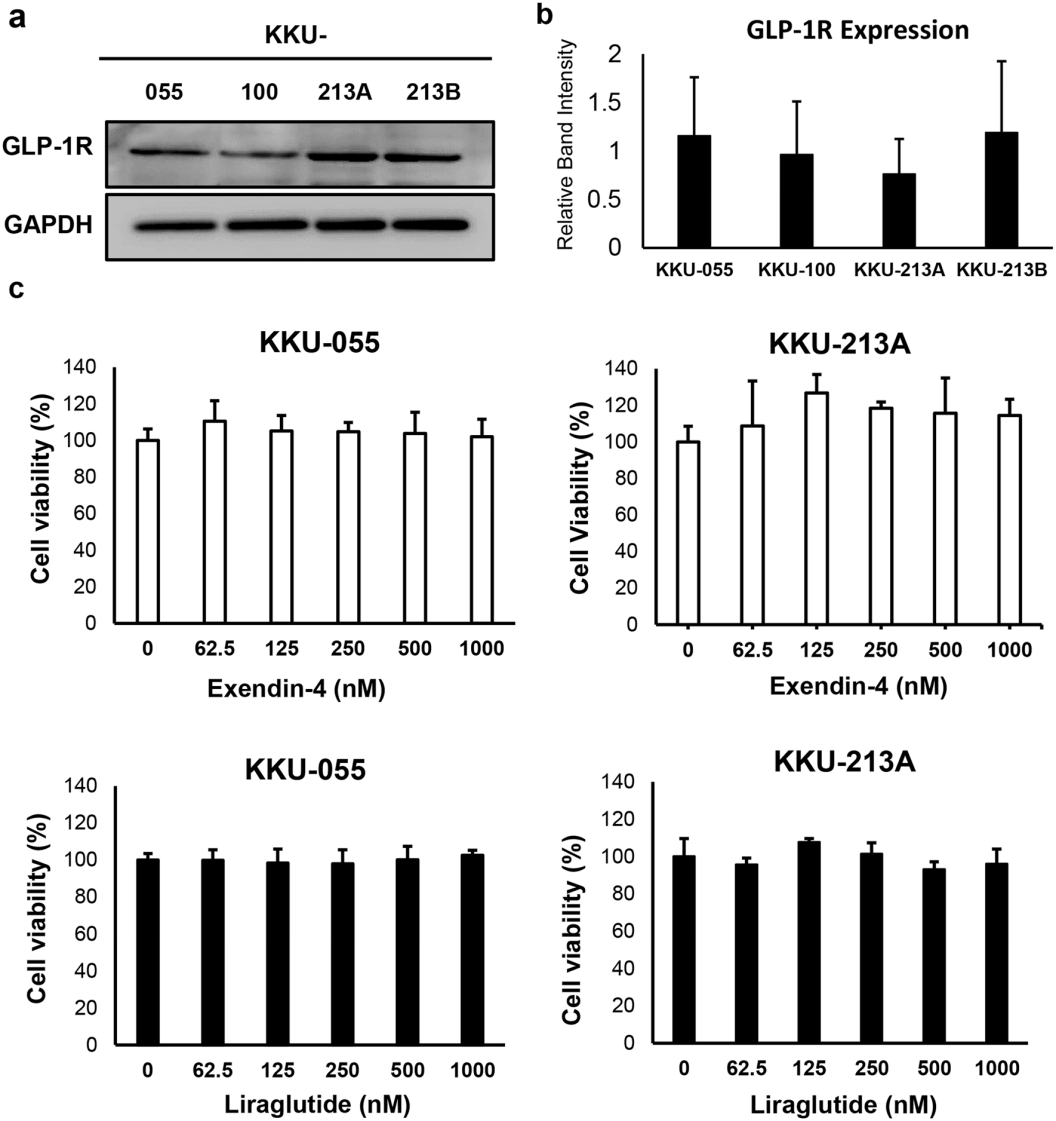
Effects of GLP-1R agonist on iCCA cell proliferation. (**a**, **b**) All examined iCCA cell lines express GLP-1R proteins without a significant difference in levels. (**c**) Neither exendin-4 nor liraglutide, two different GLP-1R agonists, show any effects on CCA cell proliferation. Western blots are representatives of 3 biological replications, and the graph represents the average band intensities from 3 biological replications. Protein expressions are normalized by GAPDH.

To investigate the roles of GLP-1R agonists in the proliferation of iCCA, in vitro experiments using iCCA cell lines were performed. Firstly, the expressions of GLP-1R protein in CCA cell lines were confirmed by Western blot, and the results showed that all tested iCCA cells expressed GLP-1R. The levels of GLP-1R expressions were not different among the 4 cell lines (Fig. 2a, b). Thus, KKU-055 and KKU-213A, 2 iCCA cell lines established from different histological grading of iCCA, were selected for further study.

Effects of GLP-1R agonist on iCCA cell proliferation were then assessed. iCCA cells were treated with exendin-4 and liraglutide, two GLP-1R agonists with different degrees of analogy to the native GLP-1. Neither exendin-4 nor liraglutide exerted significant effects on iCCA cell proliferation up to the concentration of 1000 nM (Fig. 2c). As liraglutide has a more analogous structure to the native GLP-1 and has never been studied for its effects on iCCA, it was selected for further experiments as a representative of the GLP-1R agonist.

### Liraglutide reduced migration ability of iCCA cells

**Figure 3:**
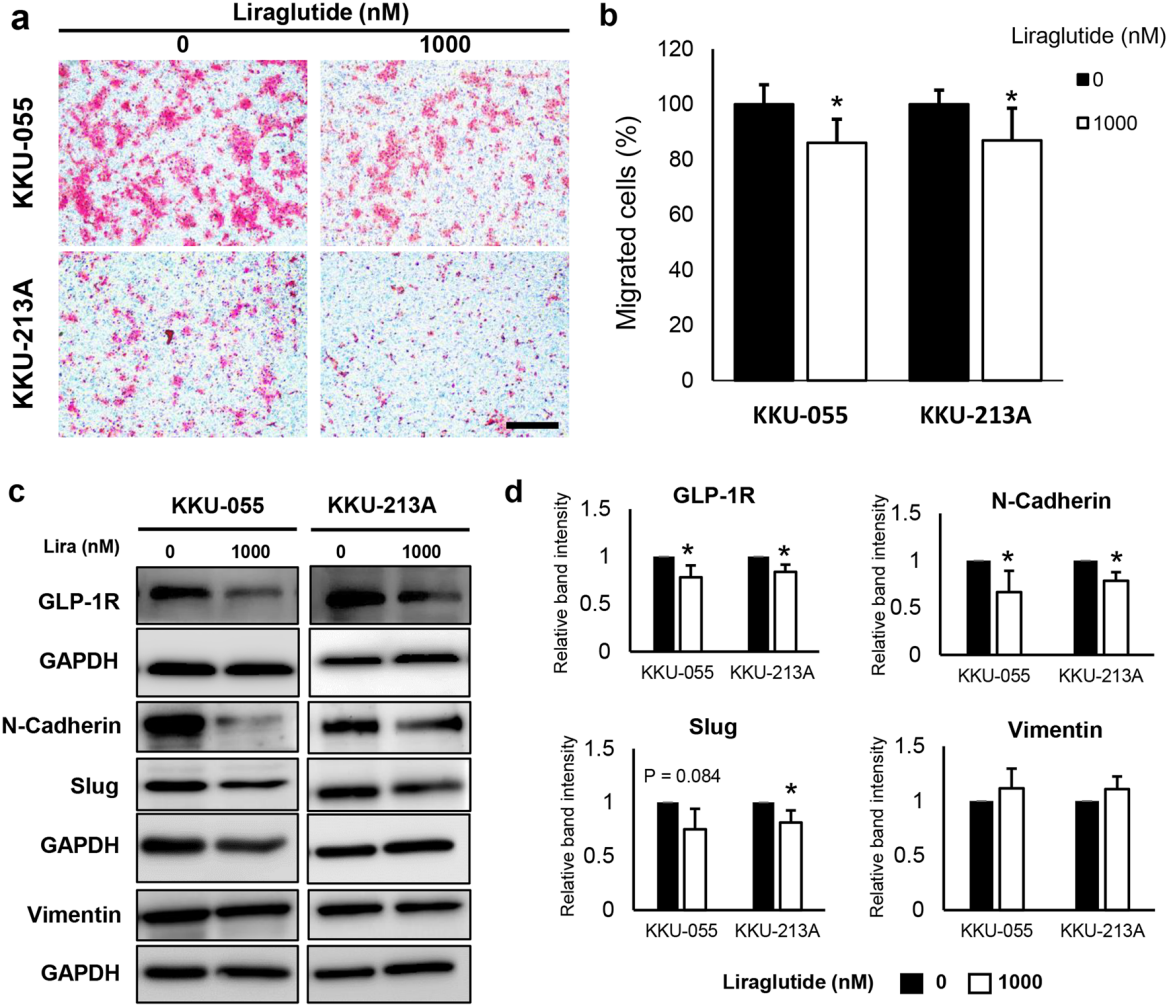
Liraglutide suppresses the migration of CCA cells by suppressing the expression of GLP-1R and epithelial-mesenchymal (EMT) markers. (**a**, **b**) Liraglutide significantly suppresses the migration of iCCA cells in both KKU-055 and KKU-213A. (**c**, **d**) The expression of GLP-1R and the EMT markers were correspondingly decreased in liraglutide-treated iCCA cells. Western blots are representatives of 3 biological replications, and the graphs represent the average band intensities from 3 biological replications. Protein expressions are normalized by GAPDH, in which the expression in the control groups is assigned as the factor of 1. Scale bars represent 100 µm. (*P < 0.05, Student’s *t*-test).

While there was no effect of GLP-1R agonist on iCCA cell proliferation in vitro, the previous study, however, showed that exendin-4 could significantly suppress the migration ability of non-*Ov*-associated CCA cells^24^. The effects of liraglutide on the migration of iCCA cells were then also examined. Liraglutide significantly suppressed the migrations of iCCA cells (*P* < 0.05) for both KKU-055 and KKU-213A (Fig. 3a, b) to a similar extent.

### Liraglutide downregulated GLP-1R expression in iCCA cells and inhibited epithelial-mesenchymal transition

A previous study demonstrated that silencing GLP-1R expression using siRNA significantly reduced the migration of CCA cells, whereas the overexpression of GLP-1R enhanced CCA cell migration^25^. Whether liraglutide affected GLP-1R expression and led to reduced migration was then investigated. Liraglutide significantly suppressed the expression of GLP-1R proteins in both KKU-055 and KKU-213A together with the reduction of epithelial-mesenchymal transition (EMT) of iCCA cells as evidenced by downregulating the expression of mesenchymal markers, namely N-Cadherin and Slug (Fig. 3c, d). However, the expression of vimentin, another examined mesenchymal marker, was not different between the control and iCCA cells treated with liraglutide.

### Liraglutide suppressed Akt and STAT3 signaling in CCA cells in vitro

**Figure 4:**
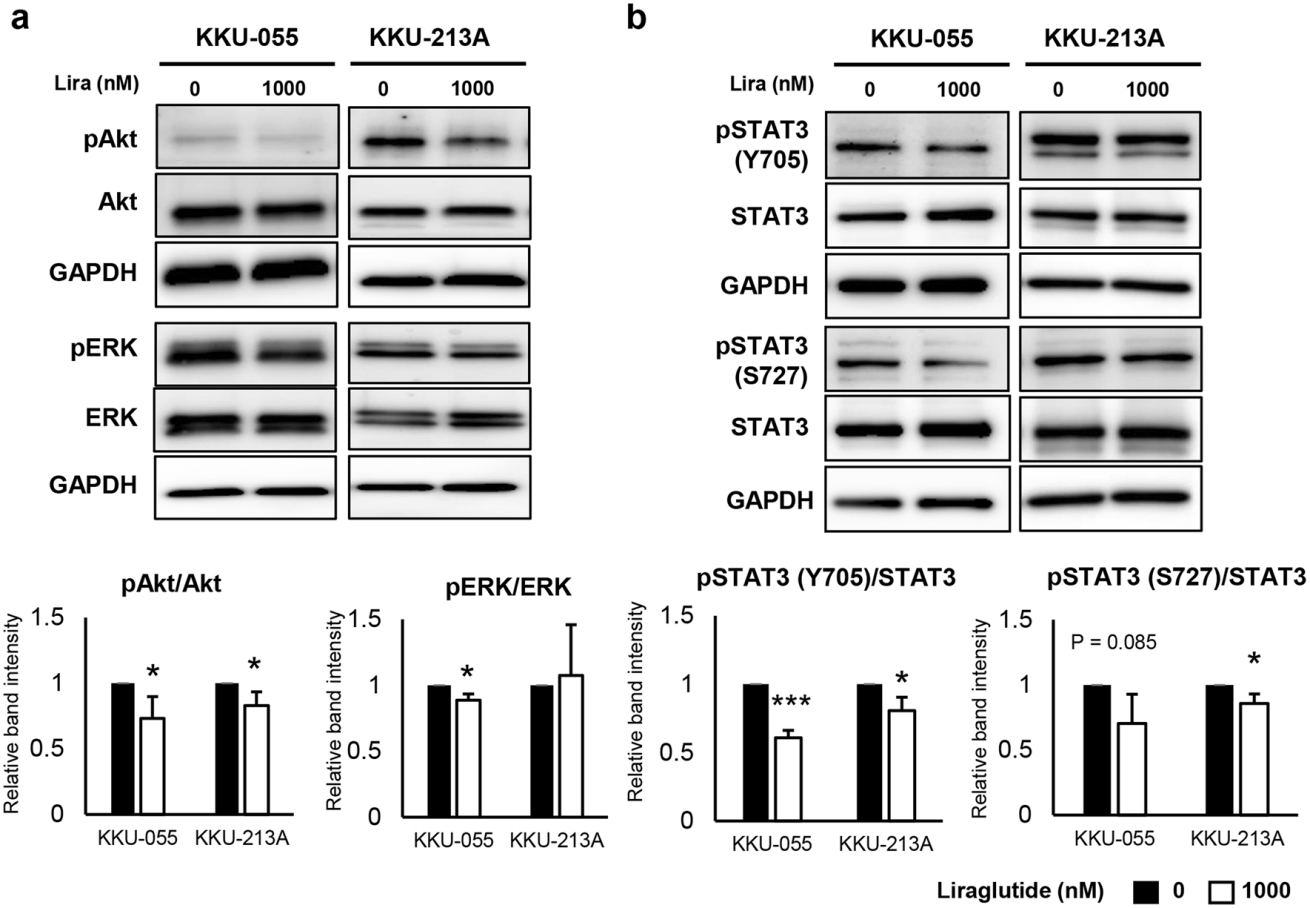
Liraglutide inhibits Akt and STAT3 pathways in CCA cells. (**a**, **b**) The phosphorylation of Akt and STAT3 is suppressed after both iCCA cells were treated with liraglutide. However, the ERK phosphorylation is slightly inhibited and statistically significant in only KKU-055. Western blots are representatives of 3 biological replications, and the graphs represent the average band intensities from 3 biological replications. Protein expressions are normalized by GAPDH, which the expression in the control groups is assigned as the factor of 1. (*P < 0.05, ***P < 0.001, Student’s *t*-test).

Since the EMT process in CCA can be regulated by several signaling pathways, the key molecules reported as underlying pathways of EMT promotion in CCA, i.e., Akt, ERK, and STAT3, were then investigated after the treatment of liraglutide. Liraglutide significantly inhibited phosphorylation of Akt and STAT3 in both KKU-055 and KKU-213A (*P* < 0.05), while the expressions of total Akt and STAT3 were not altered (Fig. 4a, b). The phosphorylation of ERK was slightly reduced but with statistical significance only in KKU-055, while it was not changed in KKU-213A (Fig. 4a, b).

### Liraglutide reduced growth and induced apoptosis of iCCA in vivo

**Figure 5:**
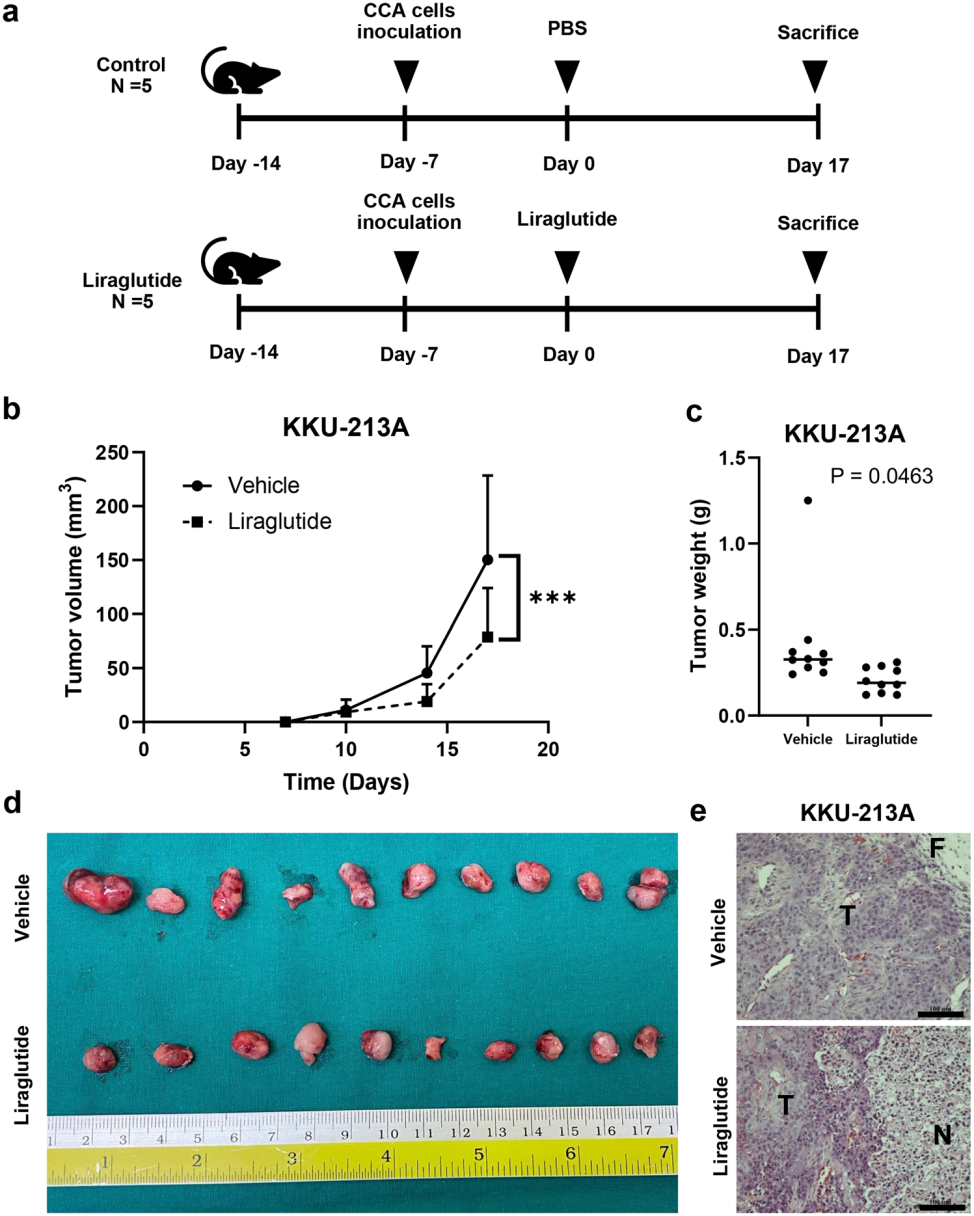
Liraglutide suppresses the growth of KKU-213A xenografts in vivo. (**a**) BALB/c Rag-2-/- Jak3-/- (BRJ) immunodeficient mice were subcutaneously inoculated with KKU-213A and then randomized into the control and liraglutide treatment groups (N = 5 mice/group). (**b**) CCA tumor volumes are significantly reduced in the liraglutide-treated group (*P < 0.001, Two-way ANOVA with Tukey’s multiple comparisons), and (**c**, **d**) the tumor weights are accordingly reduced (Student’s *t*-test). (**e**) More necrotic areas are observed in the tumors from liraglutide-treated mice. (*T* tumor cells, *N* necrotic area, *F* fibrotic area, Scale bars represent 100 µm).

The effects of liraglutide on iCCA in vivo were then investigated in a KKU-213A xenografted mouse model using the BALB/c Rag-2-/- Jak3-/- (BRJ) mice. A schematic summary of the in vivo experiments is shown in Fig. 5a. Mice receiving daily liraglutide injections showed significantly reduced tumor volume compared with the group receiving PBS injections (*P* < 0.001) (Fig. 5b). The tumor weight in the liraglutide treatment group was also significantly lower than the control group (*P* < 0.05) (Fig. 5c, d). Histological section and hematoxylin and eosin staining revealed areas of cell death inside the tumor tissues, which substantially increased in the liraglutide treatment group (Fig. 5e).

### Liraglutide inhibited multiple signaling pathways in vivo

**Figure 6:**
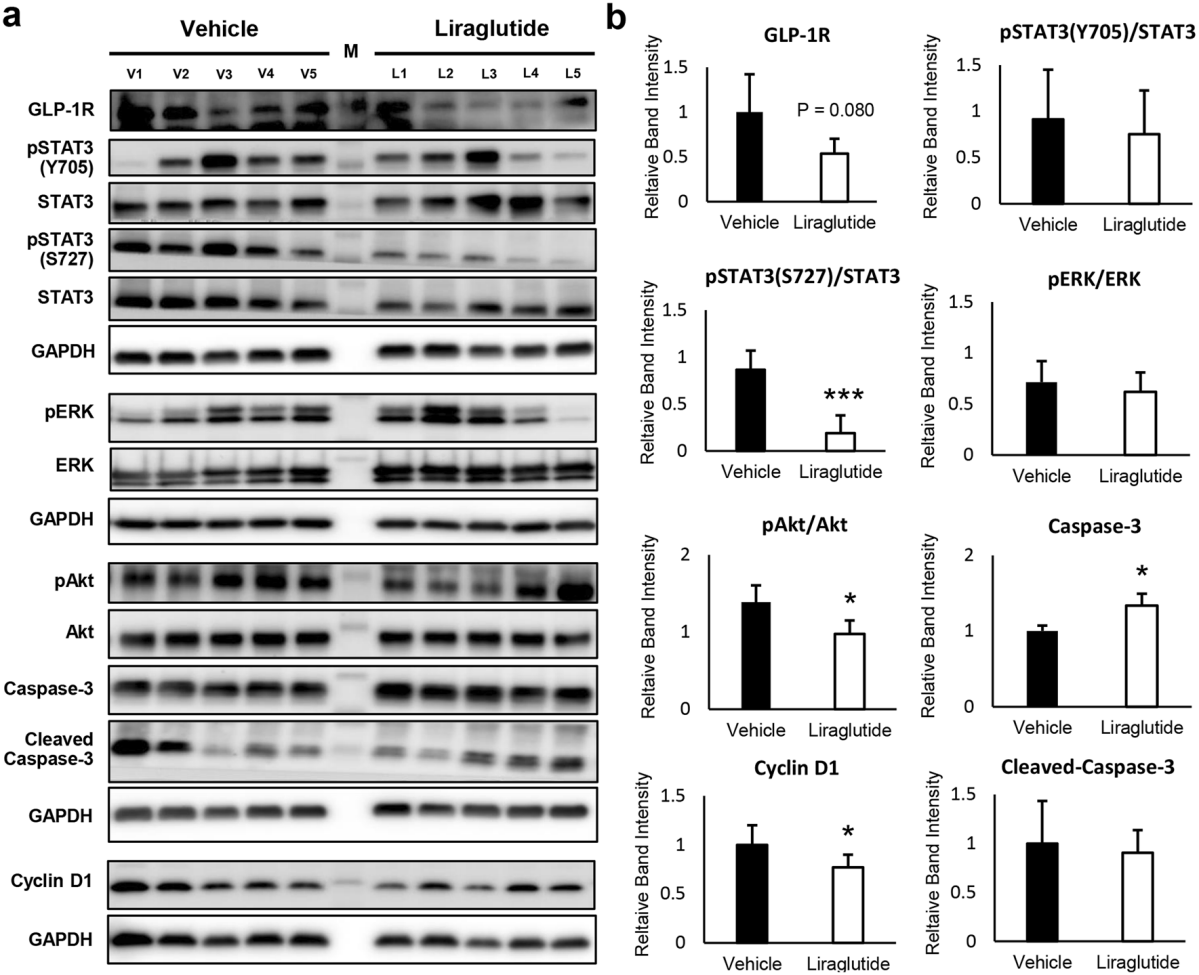
Liraglutide suppresses the expression of GLP-1R and multiple signaling pathways in vivo. (**a**, **b**) Liraglutide suppresses the expression of GLP-1R in KKU-213A xenografted tumors in vivo. It also inhibits the phosphorylation of Akt and STAT3 consistently with the in vitro experiments. The expressions of downstream targeted proteins of Akt and STAT3, for instance, apoptotic protein- caspase-3 is increased, and cell cycle regulatory protein- cyclin D1 is decreased, corresponding with the reduced tumor volumes and tumor weights in mice receiving liraglutide. Each lane of Western blot is from a xenografted tumor in one mouse, where M is the lane loaded with molecular weight marker. (*P < 0.05, ***P < 0.001, Student’s *t*-test).

The effects of liraglutide on the expressions of GLP-1R and the related molecules in xenografted tumors were explored by Western blotting (Fig. 6a, b). The expressions of GLP-1R in mice receiving liraglutide tended to be decreased with a marginal statistical significance (*P* = 0.080). The phosphorylation of Akt and STAT3 at S727 was significantly reduced in the liraglutide-treated group (*P* < 0.05), whereas ERK phosphorylation was not different. The expression of cyclin D1 was significantly decreased; on the other hand, the expression of caspase-3 was slightly increased. However, the expression of cleaved caspase-3 was not different in the treatment and control groups.

## Discussion

Associations between DM and CCA carcinogenesis and progression have been previously reported in several studies, both at the epidemiology and molecular levels^6,7,12,16,17,27^. The underlying mechanisms are known to involve the effects of hyperglycemia and probably the effects of hormonal disturbances^16,17,30,31^. Our previous reports demonstrated that high glucose level is a promoting factor for CCA via the activating effects on multiple signaling pathways^16–18^. However, where the effects of exogenous insulin on CCA development and progression remain controversial^31^, some anti-diabetic medications, e.g., metformin, show potential effects on the reduction of risk of CCA development and tumor aggressiveness^32,33^. Another anti-diabetic drug group that has emerged as a relatively new link in the DM and CCA association is incretin-based therapy, especially GLP-1R agonist^30^. Previous epidemiological studies report controversial results on the use of incretin-based therapy and the risk of CCA in different regions^21–23^. The results at epidemiological and molecular levels were not consistent, and the reported roles of GLP-1 and GLP-1R agonists in CCA are diverse. The present study was thus carried out to address the roles of GLP-1R and its agonist using in vitro and in vivo models, as well as iCCA tumor tissues obtained from patients in high mortality areas for DM and CCA. In addition, iCCA tissues and cell lines used in the study were derived from the patients in an endemic liver fluke area, which have a different molecular signature from other areas^8–11^.

In the present study, expression of GLP-1R in tumor tissues is possibly correlated with unfavorable clinical features of iCCA pateints. GLP-1R expression was significantly associated with poor histological grading. However, GLP-1R expression levels were not associated with DM status and not associated with the overall survival of the patients. This finding is consistent with the findings in Chinese patients^34^. As the overall survival of patients could be influenced by several confounding factors, we thus investigated the roles of GLP-1R agonists on cell proliferation and migration of iCCA cell lines. Although all CCA cell lines expressed GLP-1R, the treatment of GLP-1R agonists, neither exendin-4 nor liraglutide, affected the proliferation of iCCA cells. Since liraglutide has a higher analogous structure to the native GLP-1 and exendin-4 has been investigated in non-liver fluke-associated CCA^35^, only liraglutide was then selected for further experiments. Although high GLP-1R expression might be associated with metastasis of CCA^25^, the treatment of liraglutide showed the opposite results, as migration of iCCA cells was significantly inhibited after liraglutide treatment. Along with reduced migration activity, the expressions of mesenchymal makers in iCCA cells treated with liraglutide were also suppressed. The previous report by Chen et al. demonstrated that the GLP-1R itself is responsible for the migration of CCA cells^25^. Silencing GLP-1R results in reduced migration, and overexpression of GLP-1R had the opposite effect of increasing the migratory activity of CCA cells via the EMT process. In the current study, the expression levels of GLP-1R after liraglutide treatment were examined, and it was found that liraglutide treatment induced downregulation of GLP-1R in iCCA cells. These results suggested that although liraglutide can activate the GLP-1R, iCCA cells might compensate for the action of GLP-1R agonist by downregulation of the receptor, like other hormone receptors. Moreover, the native GLP-1 treatment was shown to reduce the expression of GLP-1R mRNA in pancreatic β-cells^36,37^. The effects of liraglutide on downregulated GLP-1R were also found in the xenografted tumors in the mice; even the reduced levels of GLP-1R did not reach statistical significance. Downregulating GLP-1R in xenografted tumors might also result in tumor growth suppression and induction of iCCA cell death. The effects of liraglutide on reduced iCCA cell migration in vitro and reduced tumor growth in vivo might be, nonetheless, independent from the reduction of GLP-1R and directly affecting the intracellular signaling pathways. Since it has been reported that exendin-4 also exerts anti-tumor effects, namely suppressing cell proliferation and migration and inducing chemosensitivity to oxaliplatin treatment on CCA in vitro and in vivo. Nevertheless, GLP-1R expression levels have not been reported after exendin-4 treatment^24^. Further, iCCA cell proliferation was not altered after liraglutide treatment in vitro, although GLP-1R expression was suppressed. The anti-tumor effects of liraglutide in vivo in the present study are then speculated to be due to systemic effects. GLP-1 and its analogs induce pancreatic insulin secretion, resulting in controlling blood glucose at normal levels^30^. As high glucose levels promote cancer cell growth in vitro and in vivo^16,38^, the administration of liraglutide might control plasma glucose levels in the treated mice within relatively lower ranges than the control and then slow the tumor growth. In agreement with reports showing that insulin levels and GLP-1R agonists were not associated with increased risk of intrahepatic and perihilar CCA^21–23,31^, our previous study showed insulin did not enhance CCA cell proliferation in vitro^39^ and suggests that using GLP-1R agonists in iCCA patients with DM might be safe. This study also demonstrated for the first time that liraglutide suppressed GLP-1R expression in iCCA cells, both in vitro and vivo and incorporated with suppression of iCCA cell migration and reduction of tumor growth in iCCA xenografts. GLP-1R agonists might benefit patients with iCCA in terms of slowing down the progression of the disease. Regardless of whether a group of GLP-1R agonists is associated with the increased risk of CCA, the present study suggests that liraglutide is probably safe. Patients with iCCA who use liraglutide for their DM treatment might also benefit from controlling the tumor progression with liraglutide.

Liraglutide was also shown to inhibit Akt and STAT3 phosphorylation, two major pro-tumorigenic signaling pathways in CCA. Akt is a known pathway that responds to GLP-1 activating GLP-1R in pancreatic β-cells^30^. Nevertheless, GLP-1R is classified as a G-protein coupled receptor in which signals could be sent to diverse downstream pathways via adaptive proteins. In our study, we found that STAT3 is another signaling pathway that could be regulated by liraglutide both in vitro and in vivo.

This study, nevertheless, has some limitations that need to be addressed in future studies. First, whether the induction of GLP-1R’s downregulation in iCCA is a specific effect of liraglutide or the general effects of GLP-1R agonists should be investigated. The mechanisms underlying liraglutide’s effects on reduced migration and EMT of CCA cells might also be contributed by other pathways rather than GLP-1R attenuation. Second, whether the administration of GLP-1R agonist in mice with DM would result in similar anti-tumor effects on CCA xenografts needs to be examined. Third, the tumor tissues and CCA cell lines used in this study were derived from patients in liver fluke endemic areas, which could give rise to different genetic backgrounds from non-liver fluke-associated CCA. In addition, the most prevalent histological grade of CCA in the area is well-differentiated which might be different from the other region^45–47^. Studies to validate our results with non-liver fluke-associated CCA will be helpful in clarifying the effects of GLP1-R agonists on CCA progression. Fourth, the present study analyzed tumor tissues from patients with iCCA, clinical significance and roles of GLP-1R in extrahepatic CCA need to be warranted. Finally, as a result of a limited number of iCCA patients included, statistical analyses in this study might inevitably be confounded, and the interpretation of clinical significance should be cautioned. Further study in a larger independent cohort of iCCA will help strengthen our findings.

## Materials and methods

### CCA tissues

Histologically proven paraffin-embedded CCA tissues were derived from iCCA patients admitted for surgical resection at Srinagarind Hospital, Khon Kaen University, Thailand, and archived at the biobank of Cholangiocarcinoma Research Institute, Khon Kaen University. The clinical and laboratory data were obtained from medical records. Written informed consent was obtained from all patients before collecting specimens and related data. The study protocol was reviewed and approved by The Khon Kaen University Ethics Committee on Human Research based on the declaration of Helsinki and ICH-Good Clinical Practice Guidelines (Reference No. HE661097, Approval Date: 26 February 2023).

### Immunohistochemistry

Immunohistochemical staining was performed to investigate the levels of GLP-1R expression. The clinicopathological characteristics, i.e., sex, age, subtypes of CCA, histological differentiation, vascular invasion, tumor size (longest diameter), regional lymph node metastasis, liver metastasis, and TNM stages, were included. The median age of subjects (56 years) was used as a cut-off point for the higher or lower age groups. The included laboratory parameters were preoperative fasting blood glucose (FBG), carbohydrate antigen 19-9 (CA19-9), and carcinoembryogenic antigen (CEA). After rehydration with gradient ethanol solutions, antigen retrieval was done by heating the samples in 0.1 M citrate buffer pH 6.0 in a pressure cooker for 5 min. Endogenous peroxidase activities were blocked using 3% H_2_O_2_ in methanol, and then non-specific antigens were blocked using 3% fetal bovine serum (FBS) (Gibco, Carlsbad, CA). The specimens were incubated overnight with the primary antibody against GLP-1R (1: 50) (ThermoFisher, Waltham, MA) at room temperature, then further incubated with EnVision + System-HRP conjugated secondary antibody (Dako, Carpinteria, Denmark) and the signal developed using diaminobenzidine with counterstaining using Mayer’s hematoxylin. The expression levels of GLP-1R were evaluated using the H-score system where H-score = ∑ [Intensity x frequency (%)]. The grading of GLP-1R intensity was modified from the guideline for grading membranous-expressed HER2 as recommended by the College of American Pathologists (CAP)^40^ and defined as low (+ 1), moderate to high (+ 2), and moderate to high with membranous staining pattern (3 +). Membranous staining was defined only when the basolateral aspects of the cell membrane were stained^40^. The mean of the H-score (143) was used as a cut-off point for the high and low GLP-1R expression groups. The assessment was done by two researchers under the supervision of a senior pathologist.

### Hematoxylin and eosin staining

Formalin-fixed paraffin-embedded tumor tissues were cut into 6-µm-thick slices and mounted onto glass slides. The tissues were stained with hematoxylin (Polyscience Inc., Warrington, PA, USA) for 40 s and with eosin (Sigma-Aldrich) for 30 s. The tissue sections were examined under a light microscope (Nikon, Tokyo, Japan) after mounting with Permount mounting medium (Fisher Scientific, Miami, FL, USA)^41^.

### iCCA cell lines and cell culture

iCCA cell lines, KKU-055 and KKU-213A, were established from the resected tumors of Thai patients with *Ov*-associated iCCA. Both cell lines were obtained from the Japanese Collection of Research Bioresources Cell Bank, Osaka, Japan^42^. Cells were cultured in Dulbecco’s Modified Eagle Medium (DMEM) (Gibco), supplemented with 10% FBS (Gibco), and 1% antibiotic–antimycotic (Gibco), a 37 °C, 5% CO_2_, humidified incubator. Cells were subcultured every 3 days or when they reached 80% confluence.

### Proliferation assay

Effects of GLP-1R agonists on iCCA cell proliferations were assessed using MTT assay. iCCA cells (2 × 10^3^ cells/well) were seeded into 96-well plates in a triplicated manner and incubated overnight. Then, the old media were replaced with media containing various concentrations of exendin-4 (Med Chem Express, Monmouth Junction, NJ) or liraglutide (Med Chem Express). Cells were incubated with specified GLP-1R agonists for 72 h and then 3-(4,5-dimethylthiazol-2-yl)-2,5-diphenyltetrazolium bromide (MTT) (Invitrogen, Carlsbad, CA) was added to the culture media to the final concentration of 0.5 mg/mL, and further incubated for 4 h. Formazan crystals were then solubilized with dimethyl sulfoxide and the OD540 was measured using a microplate reader (TECAN, Männedorf, Switzerland).

### Transwell migration assay

To investigate the effects of GLP-1R agonist on the migration of iCCA cells, migration assays using 0.8 µM-pore Transwell (Corning, Corning, NY) were performed. iCCA cells (4 × 10^4^ cells/wells) were seeded in the upper chamber containing 1000 nM liraglutide in serum-free DMEM in which DMEM with 10% FBS as a chemoattractant was added to the lower chamber. Cells were allowed to migrate for 12 h for KKU-055 and 6 h for KKU-213A. Non-migrated cells were removed from the upper chamber using a cotton swab, and then migrated cells were fixed in 4% paraformaldehyde at room temperature. Fixed cells were stained with 0.1% sulforhodamine B (Gibco) and washed with 1% acetic acid to remove the unstained color. Cells were photographed under the light microscope (Nikon), then the stained cells were solubilized with 10 mM Tris base solution, and the OD540 was measured and used as the migration index for CCA cell migration.

### SDS-PAGE and Western blot analysis

Primary antibodies used for Western blot analysis in this study were antibodies against GLP-1R (ThermoFisher), N-cadherin (Cell Signaling, Cambridge, MA), slug (Cell Signaling), vimentin (Cell Signaling), pAkt (Cell Signaling), Akt (Cell Signaling), pERK (Santa Cruz Biotechnology, Dallas, TX), ERK (Santa Cruz), pSTAT3 (Y705) (Cell Signaling), pSTAT3 (S727) (Santa Cruz), STAT3 (Santa Cruz), caspase-3 (Cell Signaling), cleaved caspase-3 (Cell Signaling), cyclin D1 (Santa Cruz), and GAPDH (Merck, Darmstadt, Germany).

Confluent cells were lysed using radioimmunoprecipitation assay (RIPA) lysis buffer supplemented with a protease inhibitor cocktail (Roche, Basel, Switzerland) and phosphatase inhibitor (Nacalai Tesque, Tokyo, Japan). To examine the baseline expression of GLP-1R, 1.5 × 10^5^ cells/well were seeded into triplicated wells of 6-well plates and incubated for 72 h. The effects of GLP-1R agonist on the expression of particular proteins’ expression were performed by seeding iCCA cells (2 × 10^5^ cells/well in a triplicated manner in a 6-well plate overnight and then treating the adhered cells with 1000 nM liraglutide for 24 h. Proteins from iCCA xenografted tumors were extracted by homogenization in a cold RIPA buffer supplemented with protease and phosphatase inhibitors. Total protein and protein concentrations were measured using Quick Start™ Bradford Protein Assay (Bio-Rad, Hercules, CA). Total proteins (20 µg/well) were resolved in 10–12.5% sodium dodecyl sulfate gel electrophoresis (SDS-PAGE), then electro-transferred to Polyvinylidene fluoride or polyvinylidene difluoride (PVDF) (Merck) and blocked with 5% skim milk in TBST. For multiple proteins detection in the same membranes, the membranes were cut before being hybridized with primary antibodies by guidance of apparent molecular weight markers (BLUeye prestained protein ladder, Sigma-Aldrich, St. Louis, MO) or being re-probed with different species-derived primary antibodies after being stripped off using mild stripping buffer pH 2.2 at room temperature for 30 min. Specific primary antibodies were incubated with the membrane at 4 °C overnight, and then HRP conjugated-secondary antibodies were applied for another 1 h at room temperature. The signals were developed using the Enhanced Chemiluminescent Kit (Merck) and detected using Amersham Imager 600 (GE-Healthcare Bio-Science AB, Uppsala, Sweden). Band intensities were quantitated using Image J software (National Institute of Health, Bethesda, MD).

### Xenografted mouse model

To evaluate the effect of GLP-1R agonist on the progression of iCCA in vivo, 6-week-old male BALB/c Rag-2-/- Jak3-/- (BRJ) mice were used for iCCA xenograft implantation^43^. All mice were bred and housed in the husbandry of the Northeast Laboratory Animal Center, Khon Kaen University. After acclimatization, mice were subcutaneously inoculated with KKU-213A (1 × 10^5^ cells/site of injection) in 50% Matrigel (BD Bioscience, Frankin Lakes, NJ) at both flanks. When tumor volumes reached 50–100 mm^3^, mice were randomized into the control and treatment group (5 mice/group). The treatment group received liraglutide (10 µg/kg/day) in PBS daily via the intraperitoneal injection, while the control group received the same amount of PBS daily for 17 days. The tumor volumes were measured twice a week using a digital Vernier caliper. Tumor volumes were calculated using the formula; tumor volume = (L x W2)/2, where L is the longest diameter, and W is the shortest diameter of the tumor. At the end of the experiments, all mice were euthanized using inhaled isoflurane, and open thoracotomy was used as secondary euthanasia. Tumors were snap-frozen in liquid nitrogen and kept at − 80 °C until used for Western blot analysis or fixed in 10% formalin for the histological section.

The protocols for in vivo study were reviewed and approved by the Institutional Animal Care and Use Committee (IACUC) of Khon Kaen University (Approval No. IACUC-KKU 11/66, Approval Date: 19 January 2023), based on the National Guidelines of The National Research Council of Thailand and ARRIVE guideline^44^. All mice were maintained in individual ventilation cages with a restricted environment of 12 h dark–light cycle, 23 ± 2 °C, and 30–60% relative humidity, and were allowed access to food and drink ad libitum. The body weights of mice were measured once a week, and the general health inspection was done daily by the veterinarian to evaluate the early humane endpoint.

### Statistical analysis

Continuous data was compared using two-tailed Student’s *t*-test, One-way ANOVA, or Two-way ANOVA, followed by Tukey’s multiple comparisons, regarding the assumption of each test. The categorical data was compared using Pearson’s Chi-square or Fisher’s Exact test. The normality of the data distribution was examined using Shapiro–Wilk’s test. For the bivariate correlation analysis, Pearson’s correlation coefficients were calculated using ratio data. Kaplan–Meier curve analysis and log-rank test were performed for the survival analysis. All in vitro experiments were done at least 3 independent experiments, and the data are presented as mean ± S.D. Statistical significance was assigned when *P* < 0.05. IBM SPSS Statistics Ver. 26.0.0 (IBM, Chicago, IL) and Prism GraphPad Ver. 9.0 (GraphPad, Dotmatics, MA) softwares were used for all statistical analysis.

### Supplementary Information

Supplementary Information.

## Data Availability

All data generated or analyzed during this study are included in this published article (and its Supplementary Information files).
