# Supplementary material for: Liraglutide exhibits potential anti-tumor effects on the progression of intrahepatic cholangiocarcinoma, in vitro and in vivo
Source: Sci Rep. 2024 Jun 14;14:13726. doi: 10.1038/s41598-024-64774-2 (PMC11178799; doi:10.1038/s41598-024-64774-2)
Supplement: Supplementary file 1 — Supplementary Information. [file 41598_2024_64774_MOESM1_ESM.pdf]

Supplementary data

Liraglutide exhibits potential anti-tumor effects on the progression of intrahepatic cholangiocarcinoma, in vitro and in vivo

Ronnakrit Trakoonsenathong[a, b], Waritta Kunprom[c], Chaiwat Aphivatanasiri[c, e], Padcharee Yueangchantuek[b], Paslada Pimkeeree[b, d], Supannika Sorin[b, e], Kullanat Khawkhiaw[b, e], Ching-Feng Chiu[f], Seiji Okada[g], Sopit Wongkham[b, e], Charupong Saengboonmee[a, b, e*]

[a]Cho Kalaphruek Excellent Research Program for Medical Students, Faculty of Medicine, Khon Kaen University, Khon Kaen, Thailand

[b]Department of Biochemistry, Faculty of Medicine, Khon Kaen University, Khon Kaen, Thailand

[c]Department of Pathology, Faculty of Medicine, Khon Kaen University, Khon Kaen, Thailand

[d]Faculty of Medical Sciences, Naresuan University, Phitsanulok, Thailand

[e]Cholangiocarcinoma Research Institute, Khon Kaen University, Khon Kaen, Thailand

[f]Graduate Institute of Metabolism and Obesity Sciences, Taipei Medical University, Taipei, Taiwan

[g]Division of Hematopoiesis, Joint Research Center for Human Retrovirus Infection, Kumamoto University, Japan

Send correspondence to:

Asst. Prof. Charupong Saengboonmee, M.D., Ph.D.

Department of Biochemistry, Faculty of Medicine, Khon Kaen University, Khon Kaen 40002, Thailand, Tel & Fax: +66-4336-3265, Email: charusa@kku.ac.th

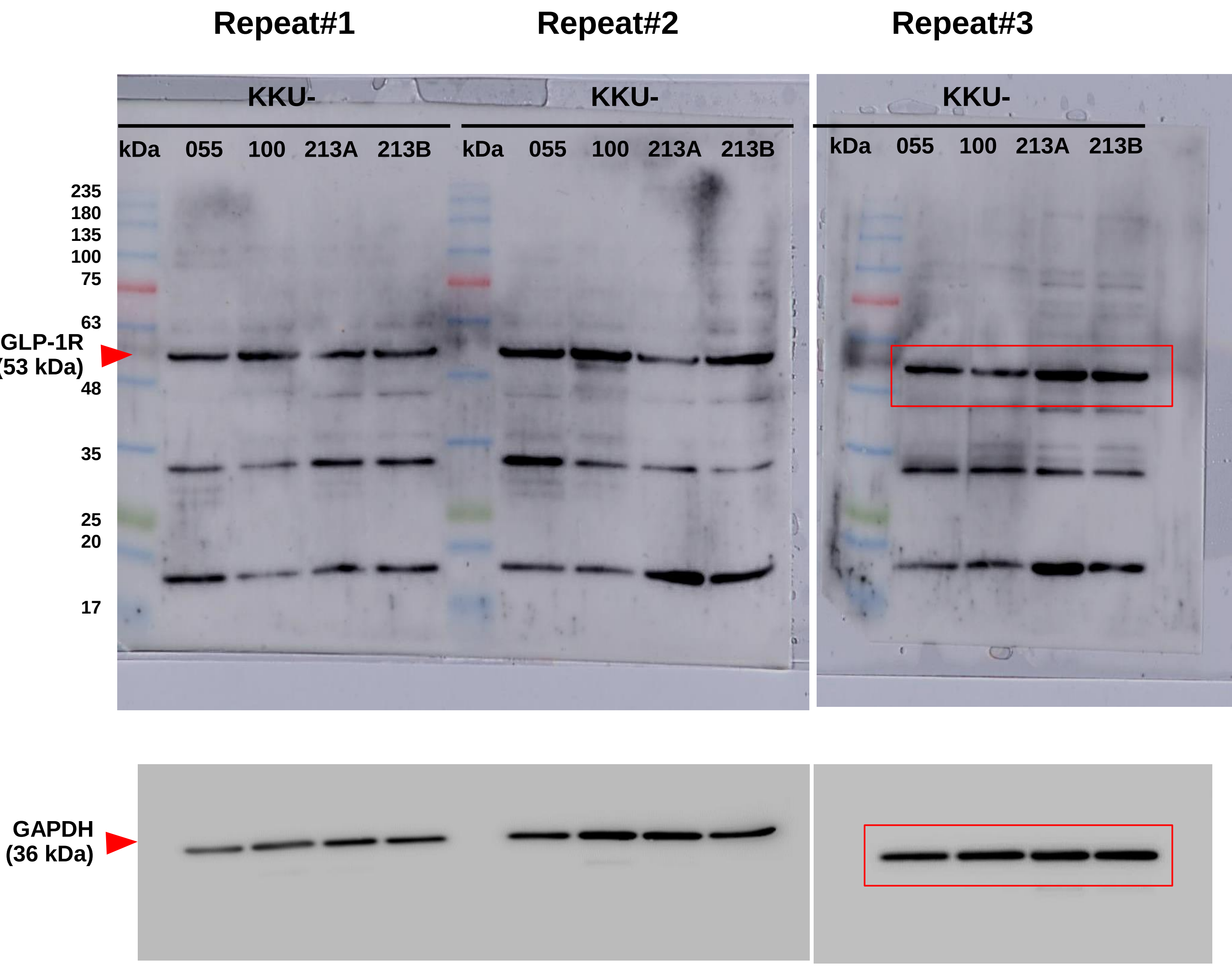

Supplementary Figure S1. Glucagon-like peptide 1 receptor expression in 4 cholangiocarcinoma cell lines.

2a. KKU-055

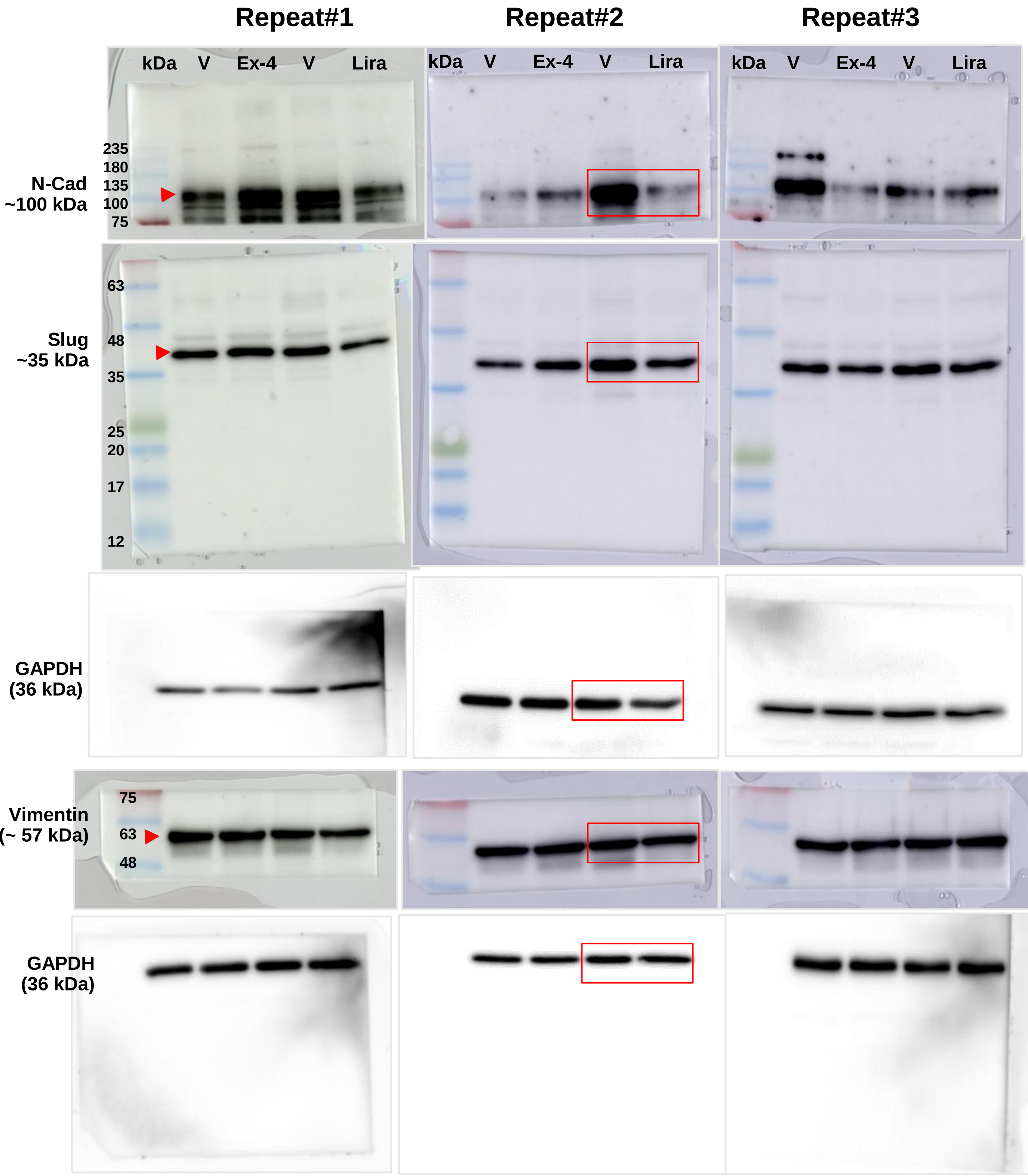

V: Vehicle control, Ex-4: Exendin-4, Lira: Liraglutide

Supplementary Figure S2. Effects of glucagon-like peptide 1 receptor agonists (GLP-1Ra) on the expressions of GLP1R and epithelial-mesenchymal (EMT) markers in cholangiocarcinoma cell lines. Liraglutide suppresses expressions of EMT markers in (a) KKU-055, and (b) KKU-213A. (c) Liraglutide suppresses expression of GLP1R. In both CCA cell lines. Ex-4: Exendin-4, Lira: Liraglutide

2b. KKU-213A

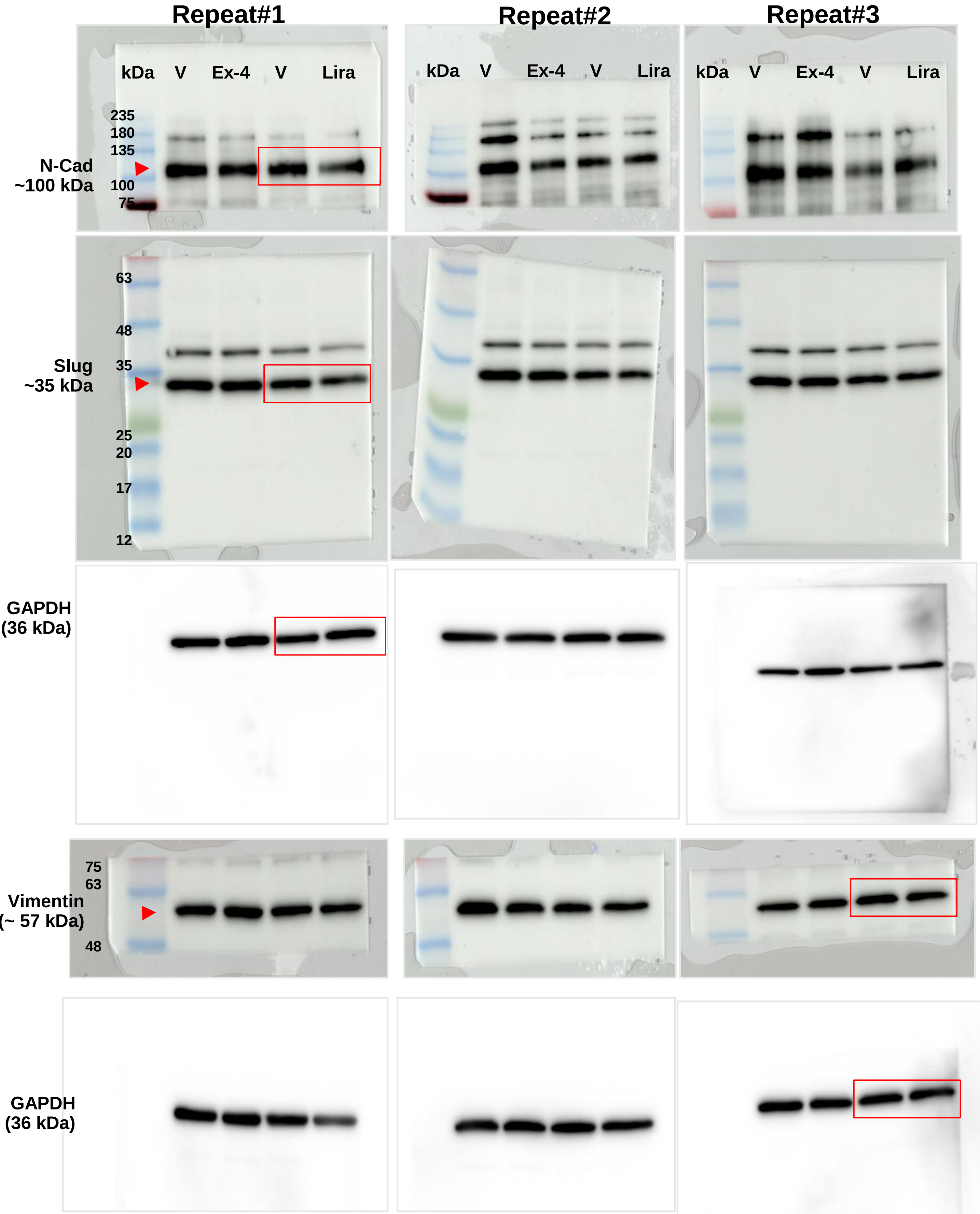

Supplementary Figure S2. Effects of glucagon-like peptide 1 receptor agonists (GLP-1Ra) on the expressions of GLP1R and epithelial-mesenchymal (EMT) markers in cholangiocarcinoma cell lines. Liraglutide suppresses expressions of EMT markers in (a) KKU-055, and (b) KKU-213A. (c) Liraglutide suppresses expression of GLP1R. In both CCA cell lines. Ex-4: Exendin-4, Lira: Liraglutide

2c

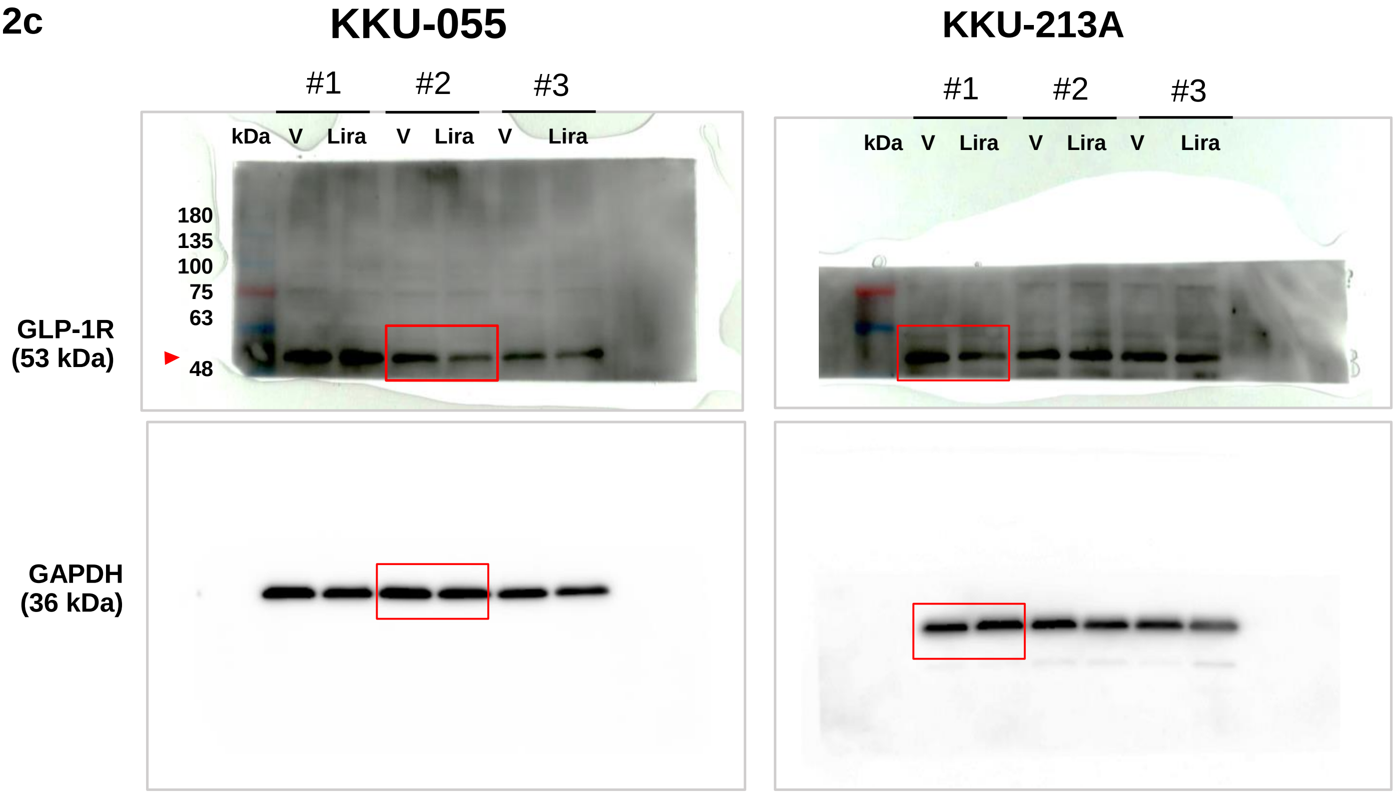

Supplementary Figure S2. Effects of glucagon-like peptide 1 receptor agonists (GLP-1Ra) on the expressions of GLP1R and epithelial-mesenchymal (EMT) markers in cholangiocarcinoma cell lines. Liraglutide suppresses expressions of EMT markers in (a) KKU-055, and (b) KKU-213A. (c) Liraglutide suppresses expression of GLP1R. In both CCA cell lines. Ex-4: Exendin-4, Lira: Liraglutide

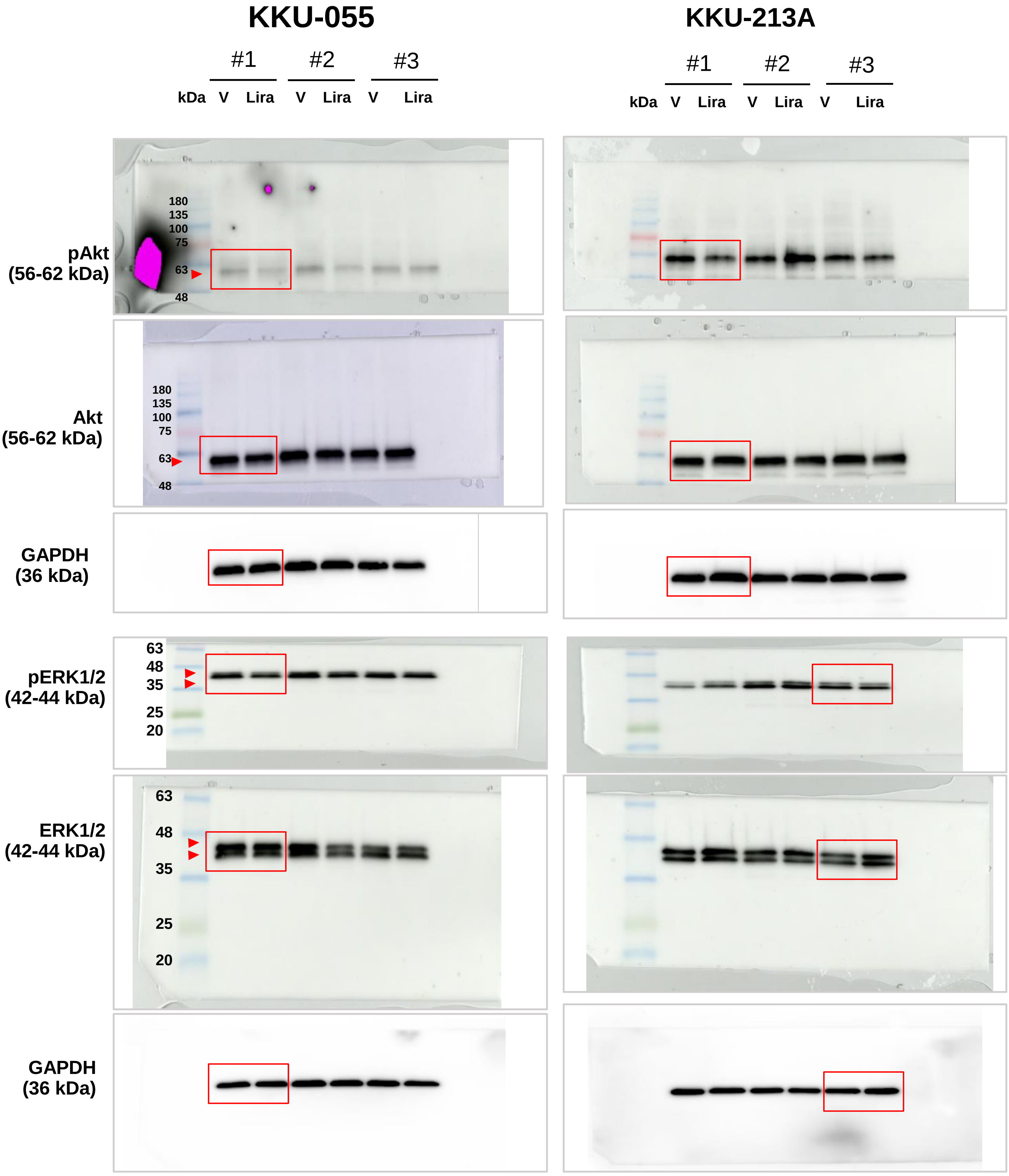

Supplementary Figure S3. Liraglutide suppresses the phosphorylation of Akt. Akt phosphorylation was suppressed after cholangiocarcinoma cells were treated with liraglutide.

4a. KKU-055

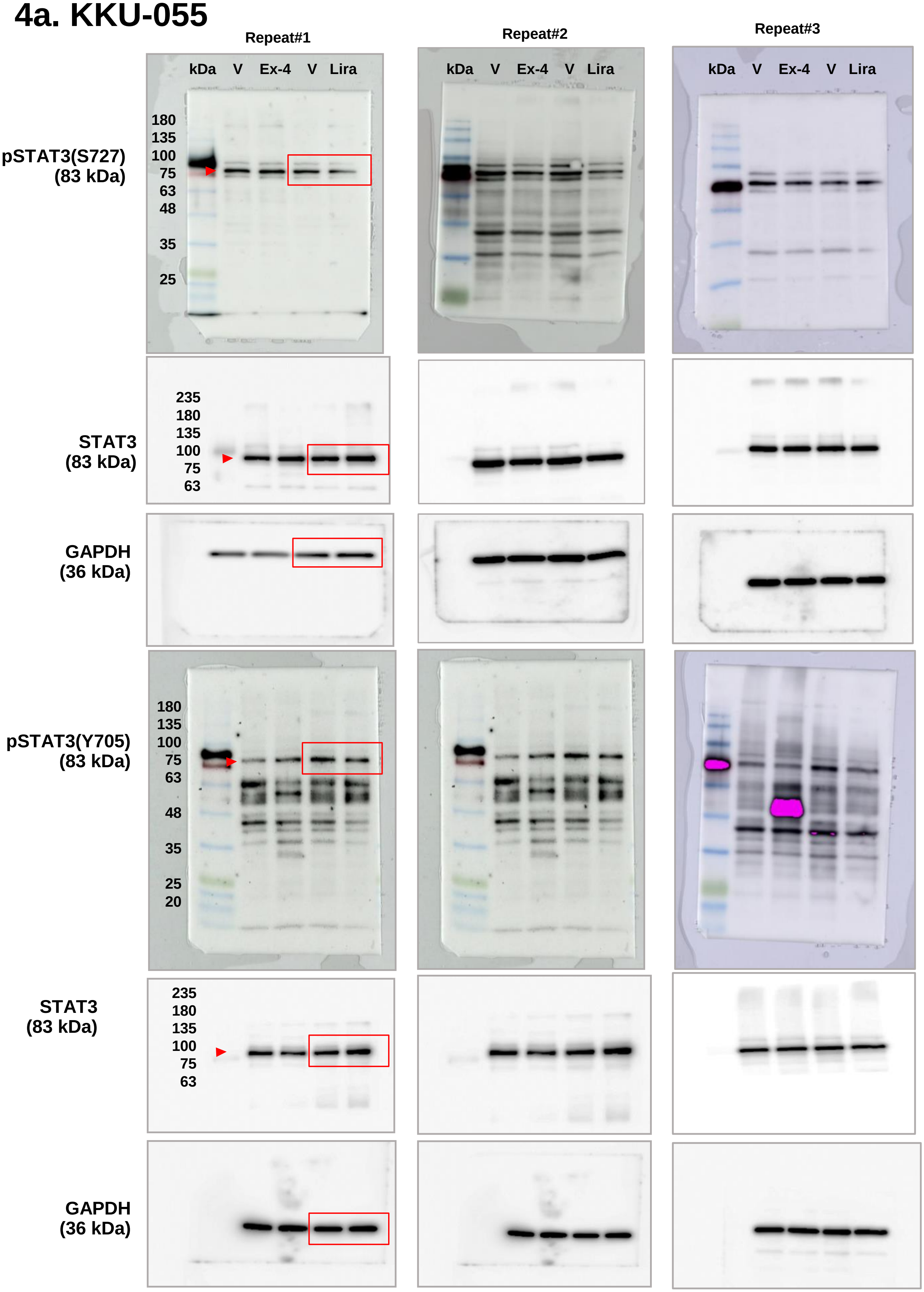

V: Vehicle control, Ex-4: Exendin-4, Lira: Liraglutide

Supplementary Figure S4. Liraglutide suppresses the phosphorylation of STAT3. STAT3 phosphorylation was suppressed after cholangiocarcinoma cells were treated with liraglutide at both Y705 and S727 in both (a) KKU-055 and (b) KKU-213A.

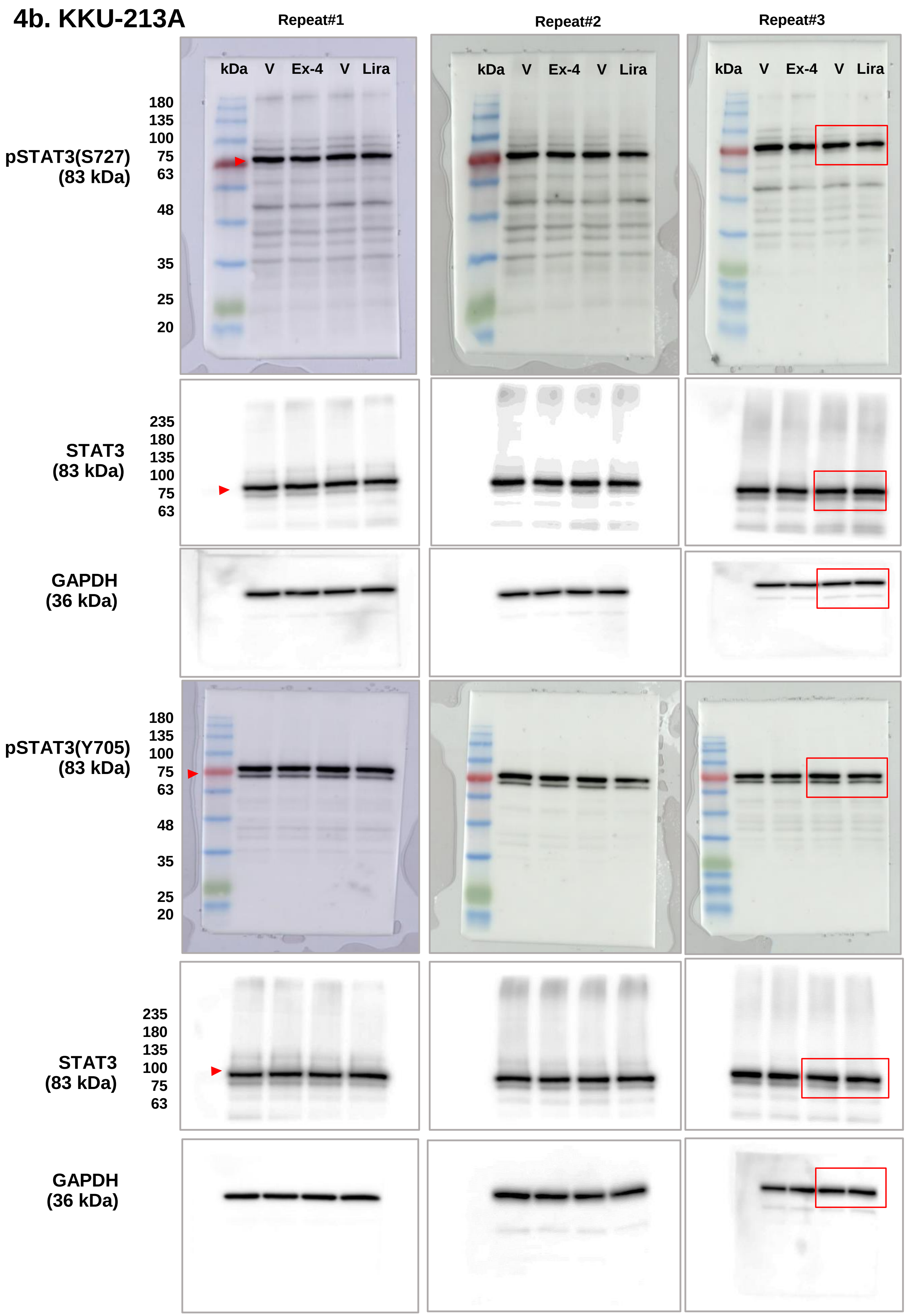

V: Vehicle control, Ex-4: Exendin-4, Lira: Liraglutide

Supplementary Figure S4. Liraglutide suppresses the phosphorylation of STAT3. STAT3 phosphorylation was suppressed after cholangiocarcinoma cells were treated with liraglutide at both Y705 and S727 in both (a) KKU-055 and (b) KKU-213A.

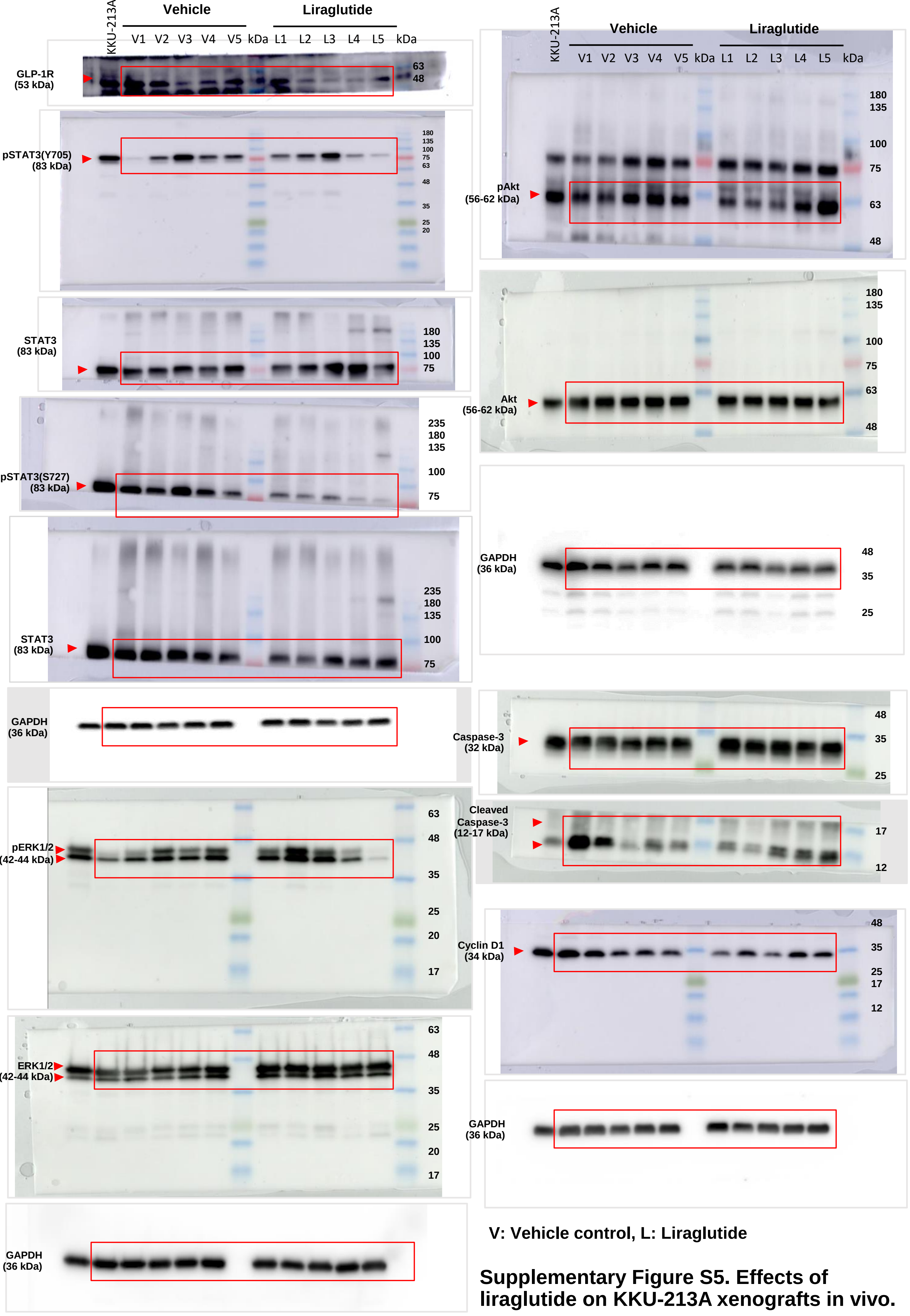

V: Vehicle control, L: Liraglutide

Supplementary Figure S5. Effects of liraglutide on KKU-213A xenografts in vivo.

Supplemental Table S1. Patients characteristics

	**Patients with CCA**
**Age (n=30), [years, median and range]**	56 [37-79]
**Preoperative FBG (n=25), [mg./dL, mean ± SD, range]**	145.48 ± 69.35 [72.00 – 346.00]
**Diabetic status (n=25), [%]**	
Diabetic-range preoperative FBG	13 [52]
Normal preoperative FBG	12 [48]
**Sex (n=30), [%]**	
Male	19 [63.30]
Female	11 [36.70]
**Histological Grading (n=24), [%]**	
Well differentiated	16 [66.70]
Moderately differentiated	6 [25.00]
Poorly differentiated	2 [8.30]
**Vascular invasion (n=29), [%]**	
Positive	23 [79.30]
Negative	6 [20.70]
**Tumor longest diameter (n=28), [cm, mean ± SD, range]**	6.64 ± 4.07 [1.30-18.00]
**Regional lymph node metastasis (n=28), [n, %]**	
Positive	10 [35.70]
Negative	18 [64.30]
**Staging of CCA (n = 26) [n, %]**	
I-III	17 [65.40]
IV	9 [34.6]
**Tumor markers**	
**CA19-9 (n=12), U/ml, Median [IQR, range]**	45.45 [145.11, 3.49 - 735]
**CEA (n=12), ng/ml, Median [IQR, range]**	4.35 [6.96, 1.33-402.00]
